# Long-lasting insecticide-treated net use and malaria infections on the Kenyan coast

**DOI:** 10.1093/trstmh/trac029

**Published:** 2022-04-12

**Authors:** Alice Kamau, Moses Musau, Grace Mtanje, Christine Mataza, Philip Bejon, Robert W Snow

**Affiliations:** KEMRI-Wellcome Trust Research Programme, P.O. Box 43640-00100, Nairobi, Kenya; KEMRI-Wellcome Trust Research Programme, P.O. Box 43640-00100, Nairobi, Kenya; KEMRI-Wellcome Trust Research Programme, P.O. Box 43640-00100, Nairobi, Kenya; KEMRI-Wellcome Trust Research Programme, P.O. Box 43640-00100, Nairobi, Kenya; Ministry of Health, Kilifi County Government, P.O. Box 519-80108, Kilifi, Kenya; KEMRI-Wellcome Trust Research Programme, P.O. Box 43640-00100, Nairobi, Kenya; Centre for Tropical Medicine and Global Health, Nuffield Department of Clinical Medicine, University of Oxford, New Richards Building, Old Road Campus, Roosevelt Drive, OX3 7LG, Oxford, UK; KEMRI-Wellcome Trust Research Programme, P.O. Box 43640-00100, Nairobi, Kenya; Centre for Tropical Medicine and Global Health, Nuffield Department of Clinical Medicine, University of Oxford, New Richards Building, Old Road Campus, Roosevelt Drive, OX3 7LG, Oxford, UK

**Keywords:** age, community, health facility, long-lasting insecticide-treated net, malaria infections

## Abstract

**Background:**

A study was conducted to examine the impact of long-lasting insecticide-treated net (LLIN) use on the prevalence of malaria infections across all ages, 25 y after a trial of insecticide-treated nets was conducted in the same area along the Kenyan coast.

**Methods:**

The study comprised four community-based infection surveys and a simultaneous 12-month surveillance at six government outpatient health facilities (March 2018–February 2019). Logistic regression was used to examine the effect of LLIN use on malaria infections across all ages.

**Results:**

There was a high level of reported LLIN use by the community (72%), notably among children <5 y of age (84%). Across all ages, the adjusted odds ratio of LLIN use against asymptomatic parasitaemia in community surveys was 0.45 (95% confidence interval [CI] 0.36 to 0.57; p<0.001) and against fevers associated with infection presenting to health facilities was 0.63 (95% CI 0.58 to 0.68; p<0.001).

**Conclusions:**

There was significant protection of LLIN use against malaria infections across all ages.

## Introduction

Long-lasting insecticide-treated nets (LLINs) are the most widespread vector control measure implemented by national malaria control programmes across Africa.^1^ In recent years there have been concerns over the effectiveness of LLINs in the face of emerging vector resistance to pyrethroids and behavioural adaptations.^2–5^ Routine surveillance of vector resistance^2^ and the interrogation of equitable LLIN coverage^1,6^ are key functions of national malaria control/elimination programmes. However, there is far less use of survey and routine data on the public health impact of LLINs under changing levels of coverage and vector resistance. Since 2017, the World Health Organization (WHO) has promoted universal coverage of LLINs in areas with high disease burdens, recognizing that coverage of all household members is likely to have a greater impact on transmission than individual protection of only young children and pregnant women.^7^ However, there are few reports on the impacts of LLINs in all age groups.^8^

Twenty-five years ago, a large controlled community trial of insecticide-treated nets in Kilifi, on the Kenyan coast, demonstrated a 44% reduction in the incidence of severe malaria presenting to hospitals and a 33% reduction in all-cause mortality among children ages 1–59 months.^9^ Underlying this impact was a nine-fold reduction in indoor resting densities of *Anopheles gambiae s.l*. and *Anopheles funestus s.s*.^10^ and a 50% reduction in the infant parasite exposure rate.^11^ Three studies of the impact of insecticide-treated nets on the risks of infection have been undertaken in this area since the 1990s. In 2000, when insecticide-treated net (ITN) use was <7%, the risk of malaria infection among children ≤10 y of age was 60% lower among net users compared with non-users.^12^ Active case detection of children ages 1–6 y, surveyed for 18 months from May 2005, showed an ITN protection of 28% against the incidence of fever with a parasite density >2500 μl.^13^ Between 2009 and 2014, when LLIN use was 71%, treated net use was associated with a 32% reduction in malaria infections among children <13 y of age presenting to one health facility.^14^ Since 2013, a low allele frequency (3.3%) of the L1014S kinase insert domain receptor (*kdr)* gene has emerged as well as a low rate of phenotypic resistance to deltamethrin and permethrin (mortality 93%) in *A. gambiae. s.l*.^15,16^

Here we analyse community and health facility data from 2018 to 2019 on the impact of LLIN use against the prevalence of malaria infections across all ages, 25 y after the first trials in Kilifi County.

## Methods

### Study area

ITNs and LLINs have been provided free of charge in Kenya since 2006 using two main delivery systems, distribution to pregnant women and children at routine health services and mass household campaigns every 3 y.^17,18^ The current subnational focus is 27 counties that constitute the highest malaria burden along the coastal, western and highlands areas.^18^ In September 2017, a mass campaign distributed 874 000 nets across Kilifi County, however, the planned 2020 campaign was delayed because of the coronavirus disease 2019 pandemic until May 2021.^19^

The present study was undertaken in the southern part of the Kilifi Health and Demographic Surveillance System (KHDSS) between March 2018 and February 2019.^20,21^ This study was conducted 6–17 months after the last mass distribution of nets. The area is rural, with the predominant occupations related to subsistence farming, and rainfall is bimodal with wet (April–June) and dry (October–December) seasons. Malaria transmission is supported predominantly by *A. funestus s.s*. and *Anopheles arabiensis.*^14,22^

### Data collection

The study was comprised of four community-based surveys (May–June 2018, August 2018, October 2018 and December 2018–January 2019) and a simultaneous 12-month surveillance (March 2018–February 2019) at six government outpatient health facilities serving these communities.^20,21^ A stratified two-stage sampling technique was used where the first stage was the health facility catchment area (six sites) and the second stage was homesteads within these six sites.

At each survey round, homesteads were randomly selected and previously selected homesteads were excluded from subsequent sampling frames. Patients treated for malaria within 2 weeks in the health facility surveillance were excluded from the community surveys. During the cross-sectional surveys for each consenting participant ≥18 y of age or the parents/guardians for children, fieldworkers obtained information from the participants on their age, sex, reported LLIN use the previous night, history of reported fever in the last 24 h, where fever was defined as ≥37.5°C. In addition, fieldworkers collected finger prick blood samples for rapid diagnostic tests (RDTs; CareStart, Access Bio, Somerset, NJ, USA) at the homestead of each participant. The fieldworkers were trained to perform and interpret the results of RDTs using Kenya Medical Research Institute (KEMRI) standard operating procedures and training schedules. Refresher training on finger pricking and RDTs was also conducted every 3 months. All participants with fever and/or a positive rapid test were advised to seek treatment at the nearest health facility.

During the continuous surveillance, March 2018–February 2019, at each health facility, information was obtained on all febrile presentations (axillary temperature of ≥37.5°C or a history of fever in the last 24 h) from the study area. Each non-pregnant patient ≥6 months of age had details recorded on residency within the study area, age, sex and reported LLIN use the night before attendance. A blood sample was taken for malaria testing using an RDT (CareStart). If the RDT result was positive, the patient received appropriate treatment as per the government of Kenya guidelines for malaria case management.^23^

Data were entered electronically using laptops in the health facilities and tablets in the community-based surveys by the study team on a PHP web-based interface and data saved onto MySQL database and synchronized onto a secure server.

### Statistical analysis

To examine reported LLIN use by age and sex documented during the cross-sectional surveys, a logistic regression model adjusted for clustering of participants was used to obtain the proportions and the 95% confidence intervals (CIs). The effect of LLIN use on the risk of malaria infections was examined separately for the community-based and health facility–based surveys, overall and by age groups (6 months–4 y, 5–9 y, 10–14 y and ≥15 y). Logistic regression models were used to examine the effect of individual LLIN use on the risk of community malaria infections and fevers presenting to facilities with infections, considering clustering of participants within homesteads. In the community-based survey, the odds ratios (ORs) were adjusted for sex, site, season and fever, while for the health facility–based survey the ORs were adjusted for sex, site, distance to health facility and season. Data analysis was undertaken using Stata 17.0 (StataCorp, College Station, TX, USA).

## Results

**Figure 1.**
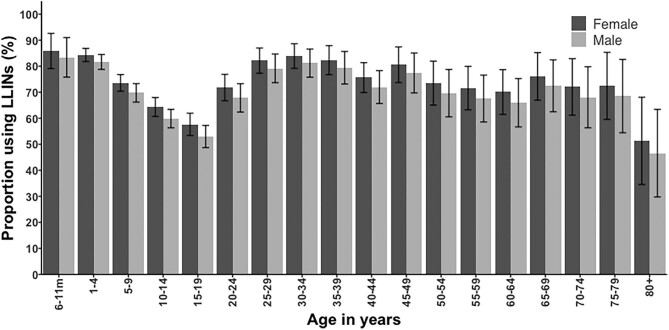
The age-specific proportion of reported LLIN use in the community stratified by sex. Ages were divided into 5-y age groups.

During the four community-based surveys, 6479 participants ages 6 months–98 y were surveyed, with 4646 (71.7%) reporting having slept under a LLIN the night before the survey. Reported individual-level LLIN use was highest among children ages 6 months–4 y (83.6%) but lower in the age groups 5–9 y (72.3%) and ≥15 y (69.9%). The lowest reported net use was among children ages 10–14 y (62.1%; Figure 1; p<0.001 for the variation by age). Across all ages, females reported sleeping under a LLIN more often than males (p=0.002) (Figure 1). There was no difference in individual LLIN use during the wet (72.3%) and dry seasons (71.0%) (p=0.24). Overall community RDT positivity was 9.9% (643/6479), lowest (6.5%) among participants ≥15 y of age and highest (13.6%) among children ages 10–14 y. The risk of malaria infections was lower among LLIN users after controlling for age, sex, site, season and fever measured during the cross-sectional survey (adjusted odds ratio [aOR] 0.45 [95% CI 0.36 to 0.57]; p<0.001; Table 1). Furthermore, significant differences were observed in the aOR in all age categories, including adults ≥15 y (aOR 0.39 [95% CI 0.28 to 0.56]; p<0.001) (Table 1).

**Table 1.** Effect of reported LLIN use on malaria infection in the community-based survey and health facility–based survey by age categories

Age group	RDT-positive LLIN users, n/N (%)	RDT-positive non-users, n/N (%)	Crude OR (95% CI)	p-Value	Adjusted OR (95% CI)	p-Value
Community-based survey						
6 months–4 y	100/1122 (8.9)	36/220 (16.4)	0.50 (0.32 to 0.79)	0.003	0.47 (0.28 to 0.79)	0.005
5–9 y	107/924 (11.6)	61/354 (17.2)	0.63 (0.43 to 0.91)	0.015	0.61 (0.41 to 0.91)	0.015
10–14 y	77/773 (10.0)	92/472 (19.5)	0.46 (0.32 to 0.66)	<0.001	0.36 (0.25 to 0.54)	<0.001
≥15 y	86/1827 (4.7)	84/787 (10.7)	0.41 (0.30 to 0.57)	<0.001	0.39 (0.28 to 0.56)	<0.001
Overall	370/4646 (8.0)	273/1833 (14.9)	0.49 (0.40 to 0.61)	<0.001	0.45 (0.36 to 0.57)	<0.001
Health facility–based survey						
6 months–4 y	2167/6363 (34.1)	461/925 (49.8)	0.52 (0.44 to 0.61)	<0.001	0.55 (0.47 to 0.65)	<0.001
5–9 y	2422/4514 (53.7)	762/1146 (66.5)	0.58 (0.50 to 0.68)	<0.001	0.62 (0.53 to 0.72)	<0.001
10–14 y	2094/3683 (56.9)	1110/1684 (65.9%)	0.68 (0.60 to 0.78)	<0.001	0.76 (0.66 to 0.87)	<0.001
≥15 y	2072/7415 (27.9)	1055/2404 (43.9)	0.50 (0.45 to, 0.55)	<0.001	0.62 (0.56 to 0.69)	<0.001
Overall	8755/21 975 (39.8)	3388/6159 (55.0)	0.54 (0.50 to 0.58)	<0.001	0.63 (0.59 to 0.68)	<0.001

Community-based survey: all age group–specific models were adjusted for sex, site, season, fever and clustering at the homestead level. Overall, the ORs were adjusted for sex, site, season, fever and clustering at the homestead level.

Health facility-based survey: all age group–specific models were adjusted for sex, site, distance to health facility, season and clustering at the homestead level. Overall, the ORs were adjusted for sex, site, season, distance to the health facility and clustering at the homestead level.

Between March 2018 and February 2019, 28 134 febrile patients 6 months–98 y of age sought treatment in one of the six outpatient health facilities from the study area. The overall proportion of reported individual LLIN use the night prior to the facility visit was 78.1%. There was no evidence of variation between individual LLIN use and seasonality (77.9% in the wet season vs 78.3% in the dry season; p=0.380). Among all febrile patients, 12 143 (43%) had a positive RDT, ranging from 59.7% (3204/5367) in children ages 10–14 y to 31.9% (3127/9819) in patients ≥15 y of age. Overall, the protection afforded by individual-level LLIN use against fever test positivity, when adjusted for sex, site, distance to health facility, age and season, was 37% (aOR 0.63 [95% CI 0.58 to 0.68]; p<0.001) (Table 1). There were differences across age, with lowest effect seen in children ages 10–14 y (aOR 0.76 [95% CI 0.66 to 0.87]; p<0.001) and highest in children <5 y of age (aOR 0.55 [95% CI 0.47 to 0.65]; p<0.001) (Table 1).

## Discussion

ITNs have been used in Kilifi County for >25 y, although at varying levels of coverage. Our study shows a high level of reported individual LLIN use by the community in 2018–2019 (72%), notably among children <5 y of age (84%). However, adolescent children have a lower reported use compared with other household members (Figure 1). The tendency towards lower LLIN use among school-aged children has consistently been shown across many settings in Africa.^24–26^ These patterns may result from a combination of factors, as most LLIN delivery programmes target LLINs to infants and pregnant women and sleeping arrangements change in crowded rural households when children reach adolescence. Reasons for lower coverage in adolescents need further investigation and approaches for increasing access, e.g. provision at schools or community-based strategies to improve rural households’ abilities to hang nets outside of traditional sleeping areas.

In Kilifi, individual LLIN use continues to provide protection against infection in the community (protective effect 48%) and against fevers associated with infection (protective effect 47%) presenting to health facilities among children ≤10 y of age. Although the approach and methods used in the present study are different, the results are similar to the reduction seen 25 y ago during the community randomized controlled trial,^11^ 21 y ago^12^ and 7–14 y ago.^13,14^ Importantly, and not previously described in the Kilifi area, are the equivalent levels of protection in the community and at health facilities in all age groups outside of childhood (Table 1). There is a growing body of evidence suggesting a large infectious reservoir among older household members that contributes to local transmission, thus all age groups should be monitored during LLIN programmes.^27–29^ Most studies consider the impact of LLIN use on infection prevalence among young children, including a recent large meta-analysis of 55 national household surveys in 24 countries^30^ and a prospective study in 5 countries.^31^ The cross-country, pooled analysis of household survey data demonstrated a 9% (95% CI 5 to 12) reduction in RDT parasite prevalence in children 6–59 months of age.^30^ The WHO-sponsored prospective study showed a pooled protective effect on infection prevalence of 37% (95% CI 22 to 59) among children ages 6 months–14 y.^31^ Both studies observed a lower protective effect against infection compared with our study in the respective age groups (Table 1). Both studies were unable to show any effect of country-level reports of pyrethroid resistance on the impact of LLINs on parasite prevalence.^30,31^ Pyrethroid resistance is emerging in Kilifi^15,16^ and the vector species composition is increasingly dominated by *A. funestus s.s*.,^14,22^ however, this has yet to translate into any declining impact on infection risks in this community. Our findings support a broader claim that LLINs in Africa continue to be valuable tools for malaria control programmes.^5^

### Caveats

The present study did not directly examine net use in the homesteads and therefore the analysis relied on reported net use rather than observed net use. The reported use may be subject to recall bias in the context of presenting for assessment of fever. Equally, we did not document the integrity and age of the nets reportedly used, which has been shown to impact the level of protection.^12,32^ Nevertheless, our approach does not differ from net reporting undertaken in national household surveys.^33^ While the community studied here was generally homogeneous (rural homestead), we did not collect data on individual and homestead-level factors related to socio-economic and educational status. It is possible that non-users of LLINs have other risk factors for malaria infection that we did not capture in this study. Finally, although we also considered reported LLIN use during the health facility surveillance, there is a potential bias in reported LLIN use since mothers are more likely to misreport (prevarication bias) their child's LLIN use because the child is sick.

## Conclusions

Routine distributions and mass household campaigns have resulted in high individual LLIN use in Kilifi. Those who report using LLINs have significant protection against malaria infections across all ages and protection against infection has been maintained for >25 y. Despite low levels of pyrethroid resistance, LLINs remain an effective and important tool on the Kenyan coast. Efforts should be made to increase sustained availability and improve use among adolescent children. Continued vector resistance surveillance should be accompanied by routine surveys at health facilities and community-based surveys on the host infection. In addition, the public health impact of LLIN use should be undertaken in all age groups.

## Data Availability

Data cannot be shared publicly because it includes homestead-level coordinates as an essential component, and these are personal identifiable data. Data that support the findings of this study are available from the KEMRI Institutional Data Access/Ethics Committee. Details of the guidelines can be found in the KEMRI-Wellcome data sharing guidelines (https://kemri-wellcome.org/about-us/#ChildVerticalTab_15). Access to data is provided via the KEMRI Wellcome Data Governance Committee (dgc@kemri-wellcome.org).
